# Is intraosseous access a safe option in adult cardiac arrest? A review of the literature

**DOI:** 10.1186/cc9714

**Published:** 2011-03-11

**Authors:** J Baombe, B Foex

**Affiliations:** 1 Manchester Royal Infirmary, Manchester, UK

## Introduction

Intraosseous (IO) cannulation for the infusion of fluids and medications was first described by Drinker and colleagues in 1922 [1]. Its use in the paediatric population has previously been validated and is now widely accepted worldwide. However, adult IO drug administration has been lagging behind for various reasons. The authors reviewed the literature to determine the feasibility and safety of this underused cannulation method.

## Methods

The MEDLINE database (1950 to week 2 August 2010) was searched using the terms intraosseous infusions, heart arrest, cardiopulmonary arrest, cardiac arrest, resuscitation, cardiopulmonary resuscitation with their appropriate combinations and truncated terms. The Embase database (1980 to week 2 August 2010) was searched using the terms intraosseous drug administration, heart arrest, resuscitation, fluid resuscitation. Both searches were limited to English language, humans and adults only.

## Results

The MEDLINE search returned 518 papers, most of them case reports not included in our final table of summary as of low level of evidence. The two studies finally included presented encouraging results but are limited by small numbers. Seventy-seven papers were found through the Embase search but none were relevant to our specific questions.

## Conclusions

IO access in adults appears to be a fast and reliable method to deliver drugs and fluids during cardiopulmonary resuscitation, allowing achievement of adequate drug concentrations and desired pharmacological responses. Despite the limited literature, it should probably be considered if other traditional methods for drug and fluid delivery have failed.
